# Linker Optimization in Lu-177 Labeled αvβ6-Integrin Binding Peptide Trimers for Targeted Radionuclide Therapy of Cancer

**DOI:** 10.1021/acs.molpharmaceut.6c00256

**Published:** 2026-05-27

**Authors:** Nghia Trong Nguyen, Tim Rheinfrank, Stefan Stangl, Falco Reissig, Susanne Kossatz, Johannes Notni

**Affiliations:** † Department of Nuclear Medicine, TUM University Hospital and Central Institute for Translational Cancer Research, (TranslaTUM), School of Medicine, 155892 Technical University Munich, Munich 81675, Germany; ‡ 662978 TRIMT GmbH, Carl-Eschebach-Str. 7, Radeberg 01454, Germany; § Institute of Pathology, School of Medicine, Technical University Munich, Trogerstr. 18, Munich 81675, Germany

**Keywords:** peptides, αvβ6-integrin, bioconjugates, radiopharmaceuticals, theranostics

## Abstract

Theranostic radiopharmaceuticals exploit the same molecular target for diagnosis and therapy. Among emerging pan-cancer targets, αvβ6-integrin is highly expressed on various malignant cell types and can be imaged clinically with Ga-68-Trivehexin. However, therapeutic αvβ6-integrin-directed radioligands remain scarce. Here, Lu-177-labeled multimerics of the cyclic nonapeptides Tyr2, sequence c­(YRGDLAYp­(*N*Me)­K), were constructed using the tetrafunctional chelator DOTPI by means of CuAAC-based and evaluated as αvβ6-integrin-targeted radiotherapeutics. PEG linkers of increasing length (PEG0, PEG3, PEG7, PEG11) were introduced between the chelator and peptides to modulate pharmacokinetics and cellular processing. The Lu-177-labeled conjugates displayed comparable polarity (log *D*_7.4_ ≈ −2) and αvβ6-integrin affinities determined by ELISA (IC_50_ ∼ 0.2–0.5 nM), indicating minimal effects of PEG length on in vitro parameters. In αvβ6-positive H2009 cells, PEG linkers markedly enhanced and prolonged receptor-mediated uptake and internalization, consistent with improved multivalent engagement. In H2009 xenograft mice, PEG linkers reduced early blood-pool activity and increased tumor uptake at 24 h, while gelofusine (4% succinylated gelatin in Ringer’s acetate) efficiently mitigated the elevated renal retention (up to 92% reduction). Performance gains plateaued beyond PEG7, identifying the PEG7 trimer (**P7**) as the preferred lead due to favorable tumor uptake/retention. **P7** exhibited high selectivity (65- to 671-fold) over other integrin subtypes, αvβ6-integrin-dependent radiotoxicity to tumor cells, and sustained tumor retention for up to 6 days according to μSPECT.

## Graphical abstract

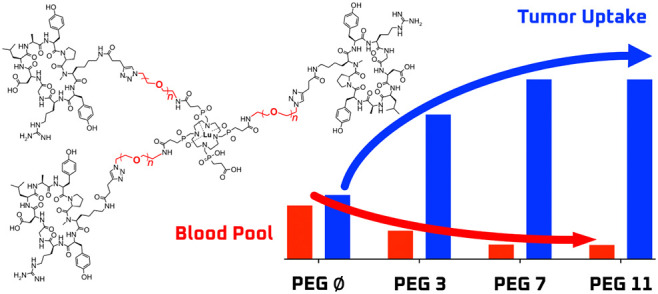

## Introduction

The term “theranostics” refers to the principle of exploiting the same biological target for diagnosis and therapy of diseases and is widely used in the context of nuclear medicine and radiopharmaceuticals.^1^ Most of the latter are radiolabeled drugs or biomolecules capable of specific target recognition, referred to as radioligands, which may be equipped with positron-emitting radionuclides for positron emission tomography (PET) imaging, such as ^18^F, ^43/44^Sc, ^68^Ga, ^61/64^Cu, or ^89^Zr;^2^ with gamma emitters for single-photon emission computed tomography (SPECT), such as ^99m^Tc, ^123^I, ^111^In, ^67^Cu, or ^203^Pb; and with alpha-emitters (^225^Ac, ^212^Pb, ^211^At) or beta-emitters (^177^Lu, ^161^Tb, ^90^Y, ^67^Cu) for targeted radionuclide therapy of cancer.^3^

A plethora of biological targets has been, and is currently being, investigated in the context of theranostics.^4^ The most impactful therapeutic approaches were those addressing the somatostatin receptor 2 (SST2, NETTER trial)^5^ and prostate-specific membrane antigen (PSMA, VISION trial),^6^ which have been the driving force behind the emergence of radiopharmaceuticals as the fastest-growing market segment in the pharmaceutical industry in recent years. Due to its nearly ubiquitous expression in the tumor microenvironment of many cancers, fibroblast activation protein (FAP) has emerged as a highly promising target for cancer imaging,^7^ and FAP inhibitors (FAPI) labeled with alpha- or beta-emitters are currently being heavily pursued as a therapeutic option for cancer patients refractory to conventional treatment lines.^4,8,9^

Among the “pan-cancer” targets with theranostic potential, the cellular surface receptor αvβ6-integrin stands out^10,11^ because of its high expression on the surface of various malignant cancer cell types,^12^ for example, pancreatic ductal adenocarcinoma (PDAC),^13^ oral squamous cell carcinoma (OSCC),^14^ ovarian^15^ and cervical cancer,^16^ and nonsmall cell lung cancer (NSCLC)^17^ and its brain metastases.^18^ Various αvβ6-integrin-targeted positron emission tomography (PET) imaging agents have been developed and applied in cancer patients,^19–27^ of which ^68^Ga-Trivehexin^28,29^ is currently the most widely used PET tracer for imaging of αvβ6-integrin expression. Its clinical potential has been demonstrated in numerous use cases, for example, in pancreatic ductal adenocarcinoma (PDAC),^30,31^ thyroid cancer,^32^ parathyroid adenoma^33^ and carcinoma,^34^ pulmonary mucoepidermoid carcinoma,^35^ as well as head-and-neck carcinoma (HNSCC)^36^ and brain metastases thereof.^37^ A prospective trial (NCT05835570) including 58 patients with nonsmall cell lung cancer (NSCLC) revealed a higher specificity (93.8%) and accuracy (91.2%) of ^68^Ga-Trivehexin PET/CT for lymph node staging than the standard-of-care, [^18^F]­FDG-PET/CT (62.5% and 64.2%, respectively).^38^ Similar findings were reported for nodal staging of colorectal, breast, pancreatic, lung, bladder, thyroid, and endometrial cancers.^39^

In light of these recent clinical developments, a therapeutic counterpart for ^68^Ga-Trivehexin is highly desirable to enable targeted radionuclide therapy for many cancers, particularly because reports on clinical use of therapeutic αvβ6-integrin ligands are scarce.^40–42^ Here we report on the progress of our ongoing efforts to develop ^177^Lu-labeled αvβ6-integrin-targeted radiotherapeutics based on cyclic nonapeptides.^43^ An investigation of tetramers of peptides with the generic sequence cyclo­(XRGDLAXp­(*N*Me)­K) (X = Tyr or Phe) revealed that the peptide Tyr2, c­(YRGDLAYp­(*N*Me)­K), is best suited for this purpose.^44^ Based on a comparison of ^177^Lu-labeled dimeric, trimeric, and tetrameric Tyr2 conjugates, we furthermore found that Tyr2-trimers offer the best compromise between target-specific uptake and nonspecific accumulation in vivo.^45^ This work aimed to investigate the effect of PEG linkers between the chelator and peptide moiety on general pharmacokinetics, target binding, and off-target uptake.

## Materials and Methods

### General

Detailed information on chemicals and materials used, lab instrumentation, and general synthetic procedures, as well as the synthesis and characterization of DOTPI­(Tyr2)_3_ and **P0**, was reported previously.^45^ All chemicals and solvents were of analytical or HPLC grade, respectively, and were used without further purification. All chemicals were obtained from Sigma-Aldrich (Taufkirchen, Germany) unless otherwise noted. DOTPI,^46^ Tyr2-alkyne,^4^ and ^nat^Ga-Trivehexin^4^ were obtained from TRIMT (Radeberg, Germany). Amino-PEG-azide linker building blocks were purchased from IRIS Biotech. Octanol–water distribution coefficients at pH 7.4 (log *D*) were determined as described previously.^20^ Synthesis and characterization of DOTPI-PEG3-triazide, DOTPI-PEG7-triazide, DOTPI-PEG11-triazide, DOTPI­(PEG3-Tyr2)_3_, DOTPI­(PEG7-Tyr2)_3_, and DOTPI­(PEG11-Tyr2)_3_ are described in the Supporting Information.

### Determination of Integrin Affinities

The protocol for determination of integrin affinities was adapted from the literature.^47^ Flat-bottom 96-well ELISA plates (BRAND, Wertheim, Germany) were coated overnight at 4 °C with the respective ECM protein (see Supporting Information, Table S13, 100 μL per well) in carbonate buffer (15 mM Na_2_CO_3_, 35 mM NaHCO_3_, pH 9.6). Each well was washed with PBS-T buffer (phosphate-buffered saline/Tween 20, 137 mM NaCl, 2.7 mM KCl, 10 mM Na_2_HPO_4_, 2 mM KH_2_PO_4_, 0.01% Tween 20, pH 7.4; 3 × 200 μL) and blocked for 1 h at room temperature with TS-B buffer (Tris-saline/BSA buffer; 150 μL/well; 20 mM Tris-HCl, 150 mM NaCl, 1 mM CaCl_2_, 1 mM MgCl_2_, 1 mM MnCl_2_, pH 7.5, 1% BSA). In the meantime, a dilution series of 6 concentrations of the test compound and an internal standard (see Supporting Information, Table S13) was prepared in an extra plate, starting from 20 μM, 4 nM, or 0.8 nM in 1:5 dilution steps (depending on the expected activity range of the test compound). Preparation of nonradioactive **P0**, **P3**, **P7**, and **P11** was done in situ according to a literature method^45^ by addition of an equimolar amount of aqueous LuCl_3_ to the respective conjugate stock solution used for further dilution, whereupon the ^nat^Lu complexes formed quantitatively within minutes.^46^ After washing the assay plate three times with PBS-T (200 μL), 50 μL of the dilution series were transferred to each well from B–G. Well A was filled with 100 μL TSB solution (blank), and well H was filled with 50 μL TS-B buffer. A solution of the respective human integrin (see Supporting Information, Table S13, 50 μL) in TS-B buffer was transferred to wells H–B and incubated for 1 h at r.t. The plate was washed three times with PBS-T buffer, and then the primary antibody (see Supporting Information, Table S13, 100 μL per well) was added. After incubation for 1 h at r.t., the plate was washed three times with PBS-T. Then, secondary peroxidase-labeled antibody (see Supporting Information, Table S13, 100 μL/well) was added and incubated for 1 h at r.t. After washing the plate three times with PBS-T, the plate was developed by quick addition of SeramunBlau (50 μL per well, Seramun Diagnostic GmbH, Heidesee, Germany) and incubated for 5 min at r.t. in the dark. The reaction was stopped with 3 M aqueous H_2_SO_4_ (50 μL/well), and the absorbance was measured at a 450 nm detection wavelength with a plate reader (POLARstar Galaxy, BMG Labtechnologies). The IC_50_ and 95% confidence intervals (CI) were calculated from all measured absorbance values (i.e., wells with different concentrations) belonging to the test compound (regularly 12 wells) as well as all values for rows A (blanks) and H (full staining), applying a sigmoidal curve fit on a logarithmic abscissa (GraphPad Prism Software). The IC_50_, LL, and UL obtained from the fit parameters were then normalized to the internal standard (see Supporting Information, Table S13).

### Radiochemistry

Radiolabeling of all compounds was performed manually, using 1 or 2 nmol peptide precursor in 300 μL of a 1 M NaOAc buffer (pH 5.9). To achieve a molar activity of 50 MBq/nmol, 50 or 100 MBq of noncarrier-added ^177^LuCl_3_ in 0.04 M HCl (Endolucin Beta, ITM, Germany) was added and heated for 30 min at 95 °C. Completion of the reaction was confirmed via radio-TLC (stationary phase: Agilent ITLC silica-impregnated chromatography paper; mobile phase: 0.1 M aqueous sodium citrate) and radio-RP-HPLC (10–90% H_2_O/MeCN + 0.1% TFA, 15 min). Radiochemical purity always exceeded 98%. Radiolabeled compounds were used for experiments without further purification and exchange of buffer; required activities were formulated in 0.9% NaCl (Pharm. Eur.).

### Stability

After radiolabeling with lutetium-177, the compounds (2–3 MBq/sample) were added to 50 μL human serum and incubated at 37 °C for 5 days. Afterward, proteins were precipitated by the addition of acetonitrile (50 μL), and the samples were centrifuged. The supernatant was transferred, and this process was repeated until no more formation of precipitate was observed. The samples were then analyzed by radio HPLC. Radiochromatograms showed no signs of decomposition.

### Cell Culture

PANC-1 (ACC 783; DSMZ; medium: DMEM) and H2009 (CRL-5911; ATCC; medium: DMEM:F12 medium with additional supplementation of 10 nM hydrocortisone, 10 nM β-estradiol, and 2 mM l-glutamine) cells were maintained in their recommended cell medium supplemented with 10% FBS and 1% Pen/Strep. All cell lines were cultivated in a monolayer at 37 °C in a humidified atmosphere with 5% CO_2_. Trypsin-EDTA (0.25% EDTA v/v) was used to harvest cells. Cell-based assays were conducted when growth reached 80% confluency. Cells were authenticated and regularly tested for the absence of mycoplasma contamination.

### Cellular Uptake Assays

αvβ6-dependent uptake of the ^177^Lu-labeled conjugates **P0**, **P3**, **P7**, and **P11** was determined in H2009 (αvβ6-positive) and PANC-1 (αvβ6-negative) cells. 100,000 cells were seeded in suspension into a 96-well V-bottom plate directly before the assay. For blocking, ^nat^Ga-Trivehexin was added at a final concentration of 250 nM 15 min prior to treatment with the radioligand. Subsequently, the ^177^Lu-labeled compounds were added at a final concentration of 0.3 nM, and the cells were incubated at 37 °C for 60 min, or for 5 min, 30 min, 1 h, 2.5 h, and 5 h for determination of uptake kinetics, respectively. After incubation, cells were pelleted at 600 × *g* for 4 min and washed twice with PBS. Cells were lysed with 1 M aqueous NaOH, and the activity in the collected cell lysates was determined using a Wizard2 automated gamma counter (PerkinElmer). The cellular uptake (membrane-bound and internalized activity) was quantified as a percentage of the total added radioactivity.

### Radiotoxicity Assay

In vitro cell viability was determined in H2009 and PANC-1 cells using the resazurin (Alamar Blue) assay under the same conditions as reported for cell culture, which measures viability and proliferation and is a valid method for determining radiation-induced cytotoxicity.^48^ **P7** was standardly radiolabeled with a molar activity of 50 MBq/nmol. H2009 and PANC-1 cells were seeded in 96-well plates and treated for 12 or 24 h with **P7** activities of 0.25, 0.5, 0.75, or 1 MBq per well and without addition of activity (untreated control). Then, the activity was washed off, and cells were incubated with fresh medium for 72 h before viability was determined using a fluorescence plate reader. In addition, control wells (1 per replicate) were incubated without the addition of radioactivity (untreated), their absorbance values set to 1 (100%), and viability values determined for treated wells were calculated as percentages of the control.

### Animal Experiments

All animal studies were performed in accordance with the National Guidelines for Animal Protection, Germany, with the approval ROB-55.2-2532.Vet_02-19-62 of the Regional Animal Care Committee of the Government of Oberbayern and were overseen by a veterinarian. Animal experiments were conducted in accordance with the guidelines of EU Directive 2010/63/EU for animal experiments using CB17/lcr-Prkdc^scid^/lcrlcoCrl severe combined immunodeficiency (SCID) mice (Charles River, Sulzfeld, Germany). For the subcutaneous xenograft model, 400,000 H2009 cells/100 μL matrigel:medium (1:1) were injected into the right shoulder of the mouse (using Matrigel from Corning, catalog #354234). Experiments were conducted 5 weeks postimplantation when the tumor size reached 5–10 mm maximum diameter. Animals were anesthetized with 2% isoflurane for all procedures. For biodistribution analysis, approximately 60–80 pmol (3–4 MBq) of **P0**, **P3**, **P7**, and **P11** (molar activity A_m_ = 50 MBq/nmol) in 200 μL of 0.9% saline were injected intravenously into the tail vein of the mouse (*n* = 4–5 per time point). 100 μL Gelofusine ISO (B. Braun, Melsungen, Germany; 4% succinylated gelatin, *M*_W_ = 30 kDa, formulated in Ringer’s acetate) was administered i.v. as a bolus, 2 min before the activity, as a kidney protective agent. The animals were sacrificed at different time points (90 min, 24 h, and 120 h after injection). Blood, relevant organs, and tumor xenografts were collected, weighed, and their radioactivity contents quantified with a Wizard^2^ automated gamma counter (PerkinElmer). Injected activity per gram tissue (%IA/g) values were calculated from measured activities and organ/tissue weights.

μSPECT imaging was performed on a nanoScan SPECT/CT (Mediso, Budapest, Hungary) at different time points (1 h, 24 h, 96 h, 144 h) after intravenous injection of approximately 26 MBq **P7;** scan time: 60 min. Gelofusine ISO (100 μL) was injected 2 min before the activity earlier to reduce kidney uptake of the radiopharmaceutical. Reconstruction, image analysis, and quantification of SPECT data were performed using Nucline and Interview Fusion software (both Mediso).

### Statistics

Statistical analysis was conducted using GraphPad Prism (10.6.1). Bar graphs typically display averages and standard deviations, unless specified otherwise. Significance was tested using Student’s *t*-test and *p*-values of <0.05 were considered significant.

## Results

### Peptide Conjugates

The bifunctional chelator DOTPI^46^ was chosen as a basis for ^177^Lu-labeled multimeric peptides because of its highly efficient radiometal ion incorporation^49^ and the presence of four chelator-independent conjugation handles.^50^ The conjugates were synthesized using a proven “click chemistry” strategy,^51^ the greatest advantage of which is that no protective groups are required on the reactants,^52^ allowing for straightforward assembly of conjugates with a multitude of functional groups.^53,54^ The approach relies on copper-catalyzed azide–alkyne cycloaddition (CuAAC) of unprotected azide- or alkyne-functionalized chelators,^55^ which, however, requires an excess of Cu^I^ (obtained by in situ reduction of Cu^II^ with ascorbate) due to the strong Cu-sequestering properties of DOTPI. Removal of the DOTPI-bound copper after CuAAC is achieved by transchelation with the stronger Cu^II^ ligand 1,4,7-triazacyclononane-1,4,7-triacetic acid (NOTA) at pH 2.2 in aqueous media, which is tolerated by most functional groups.^56^ This approach was already successfully applied to elaborate trimers^20,57^ of the ^68^Ga chelator TRAP,^58^ as well as DOTPI tetramers,^44^ trimers and dimers,^45^ and monomers of αvβ6-integrin binding peptides.^43^

Synthesis of the conjugate ^177^Lu-DOTPI­(Tyr2)_3_ was reported previously, starting from a chelator building block without PEG linkers referred to as DOTPI-triazide^45^ (see Scheme 1; PEG0). Conjugates with PEG3, PEG7, and PEG11 linkers were assembled in the same way, which required the synthesis of suitable DOTPI-PEG*n*-triazide building blocks (Scheme 1). This was done by coupling the respective 1-azido-PEG*n*-Ω-amines to DOTPI in DMSO, using HATU or PyAOP as a coupling agent according to an established protocol^45^ (see Supporting Information). However, we noted that the syntheses became less efficient with an increasing number of PEG repeats. Isolated yields were approximately 28%, 19%, and 11% for the PEG3, PEG7, and PEG11 variants of DOTPI-PEG*n*-triazide, respectively.

**Scheme 1:**
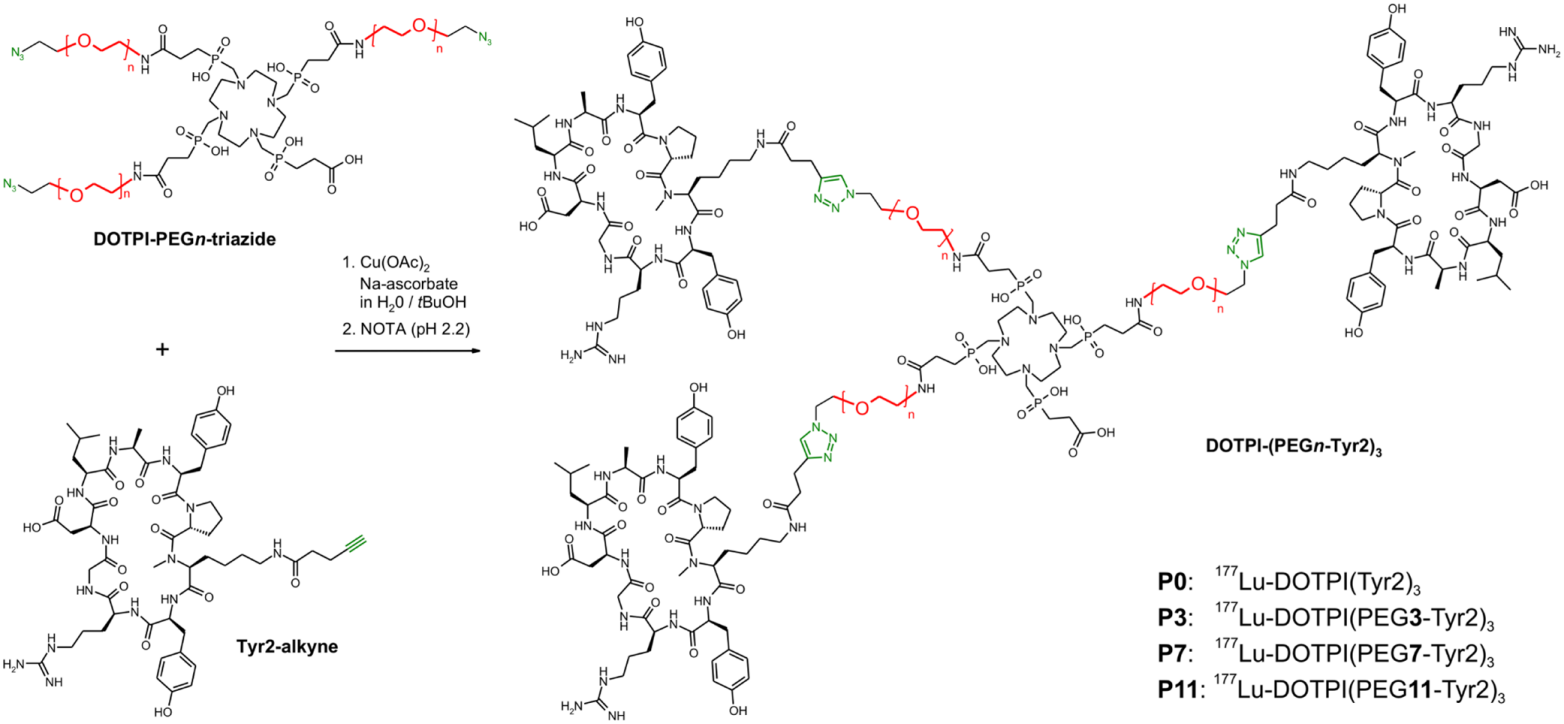
Synthesis of DOTPI Trimers of the αvβ6-Integrin Binding Peptide Tyr2-Alkyne^28^ via Cu^I^ Catalyzed Alkyne–Azide Cycloaddition (CuAAC), Followed by Cu Removal by Transchelation^51^ with 1,4,7-Triazacyclononane-1,4,7-Triacetic Acid (NOTA)^a^

a Functional groups formed by, or involved in, CuAAC are highlighted in green; PEG linkers in red.

### In Vitro Studies

The conjugates ^177^Lu-DOTPI­(Tyr2)_3_, ^177^Lu-DOTPI­(PEG3-Tyr2)_3_, ^177^Lu-DOTPI­(PEG7-Tyr2)_3_, and ^177^Lu-DOTPI­(PEG11-Tyr2)_3_ were labeled with ^177^Lu for 30 min at 95 °C in sodium acetate buffer (pH 5.9) using n.c.a. ^177^Lu^III^ in 0.04 M HCl (Endolucin Beta, ITM, Germany), which reliably afforded the radiolabeled conjugates **P0**, **P3**, **P7**, and **P11**, respectively, with radiochemical yields exceeding 98% according to radio-TLC and radio-HPLC without a separate purification step. The radiolabeled compounds were used for determination of the octanol–water distribution coefficients at pH 7.4 (log *D*_7.4_) by shake-flask. Determination of integrin affinities by ELISA was done using the nonradioactive Lu^III^ complexes of the conjugates, prepared from aqueous LuCl_3_ solution.

The PEG-free conjugate **P0** and the one with a short linker, **P3**, showed the same polarity (log *D*), while a higher number of PEG repeats in **P7** and **P11** resulted in a slightly lower hydrophilicity (Table 1). The αvβ6-integrin affinities, expressed as 50% inhibition concentrations (IC_50_), were in the same range and generally very high, albeit slightly lower for **P11**. Although some deviations in the in vitro parameters were observed, it can nevertheless be assumed that these variations are minor and cannot be considered the cause of altered binding behavior in cells or in vivo.

**Table 1:** In Vitro Data for Lu-Labeled Peptide Conjugates^a^

Code	Radiopharmaceutical	*M*_W_ [g mol^–1^]	log *D*_7.4_	αvβ6-integrin IC_50_ (95% CI)
**P0**	^177^Lu-DOTPI(Tyr2)_3_	4493.89	–2.06 ± 0.06	170 pM (90–230)
**P3**	^177^Lu-DOTPI(PEG3-Tyr2)_3_	4848.26	–2.05 ± 0.02	200 pM (79–306)
**P7**	^177^Lu-DOTPI(PEG7-Tyr2)_3_	5376.89	–1.99 ± 0.02	166 pM (74–238)
**P11**	^177^Lu-DOTPI(PEG11-Tyr2)_3_	5905.53	–1.91 ± 0.05	506 pM (270–729)

a The αvβ6-integrin affinities (IC_50_) were determined by ELISA using the nonradioactive ^nat^Lu^III^ complexes, prepared in situ by addition of a stoichiometric amount of Lu^III^ to stock solutions of the respective conjugate. Data for **P0**/^177^Lu-DOTPI­(Tyr2)_3_ were reported previously^45^ and are shown for comparison.

The αvβ6-integrin-dependent cellular uptake and internalization of **P0**, **P3**, **P7**, and **P11** were investigated using H2009 (αvβ6-positive, human lung adenocarcinoma) and PANC-1 (αvβ6-negative, human pancreatic ductal adenocarcinoma) cells. We observed that the presence and length of PEG linkers had a pronounced effect on αvβ6-integrin-mediated cell binding and internalization (Figure 1). Without PEG (**P0**), cellular uptake reached a plateau after approximately 1 h and did not further increase afterward. With increasing numbers of PEG units in compounds **P3**, **P7**, and **P11**, a higher uptake was observed after 1 h (Figure 1A), as well as a sustained increase over several hours, while the curve slopes correlated positively with the linker length (Figure 1B).

**Figure 1:**
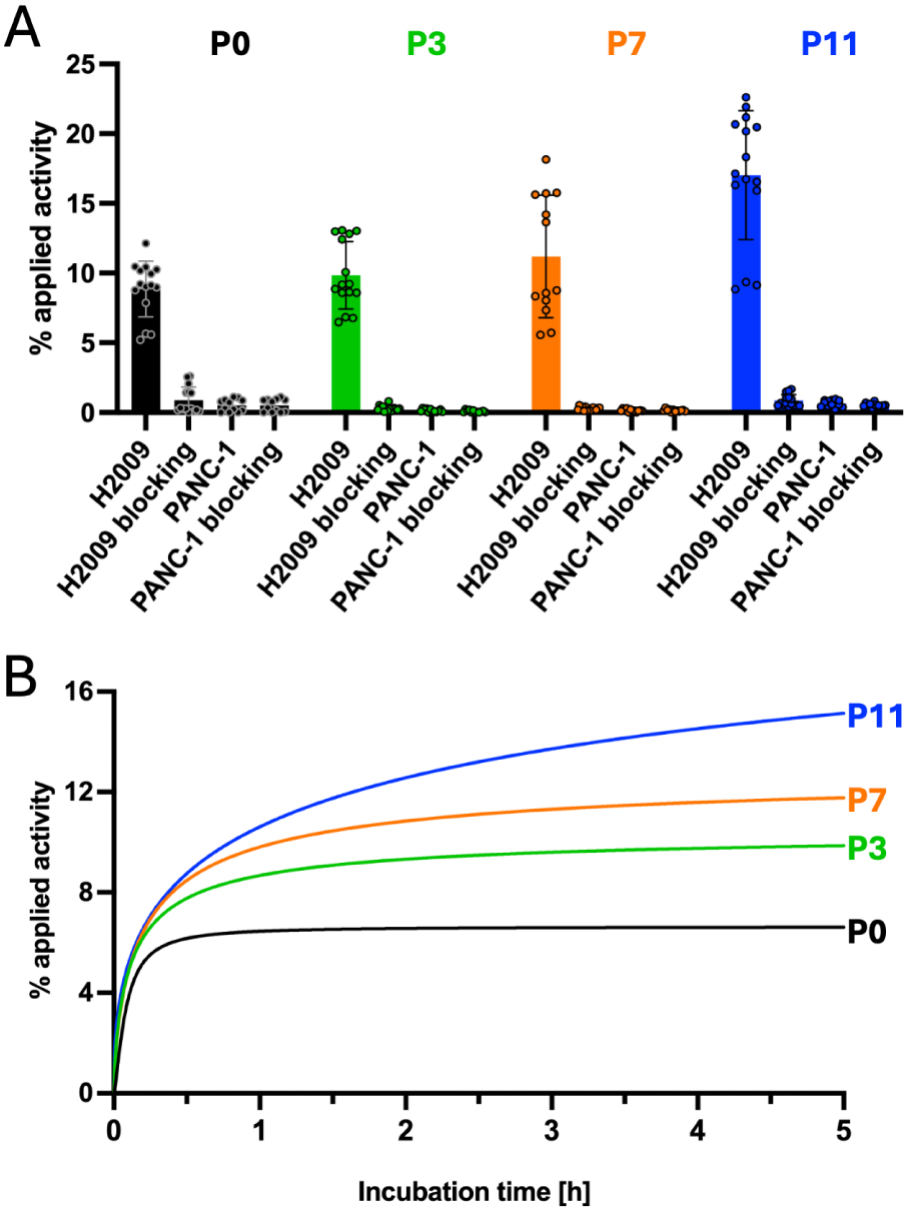
Cellular uptake characterization of **P0**, **P3**, **P7**, and **P11**. (A) Uptake of ^177^Lu-labeled conjugates (0.3 nM, 50 MBq/nmol, incubation for 1 h at 37 °C, *n* = 15) in αvβ6-integrin-expressing H2009 cells and non-αvβ6-integrin-expressing PANC-1 cells, with and without blockade (250 nM ^nat^Ga-Trivehexin, 15 min before radiolabeled compound was added). Differences between H2009 uptakes and the other data sets (PANC-1 and blockade) were significant (*p* < 0.001) for all compounds. (B) Uptake kinetics of ^177^Lu-labeled conjugates (0.3 nM, 50 MBq/nmol) in H2009 cells; 4-parameter variable slope fit curves (GraphPad Prism) to data acquired for incubation times of 5 min, 30 min, 1 h, 2.5 h, and 5 h at 37 °C (*n* = 5 per time point; for individual curves with data points, see Supporting Information, Figure S2).

### Biodistribution in Tumor-Bearing Mice

The biodistribution of all compounds was assessed in mice bearing subcutaneous αvβ6+ H2009 tumor xenografts (Figure 2). PEG linkers did not show a strong or systematic influence on nonspecific uptake in several organs and tissues (heart, pancreas, muscle, liver) but affected a lower activity concentration in blood and spleen. Although PEGs apparently led to increased kidney uptake, the kidney retention (range 156–219%IA/g) was efficiently blocked by gelofusine (up to 92% reduction for **P7**, 90 min p.i.) to reach comparable levels for all radiopeptides (range 14–28%IA/g). Particularly at later time points, PEG linkers resulted in a comparably higher uptake in the tumor, intestines, and stomach, with the latter showing a physiological αvβ6-integrin expression in mice and therefore serving as a control for target specificity of uptake.^59^

**Figure 2:**
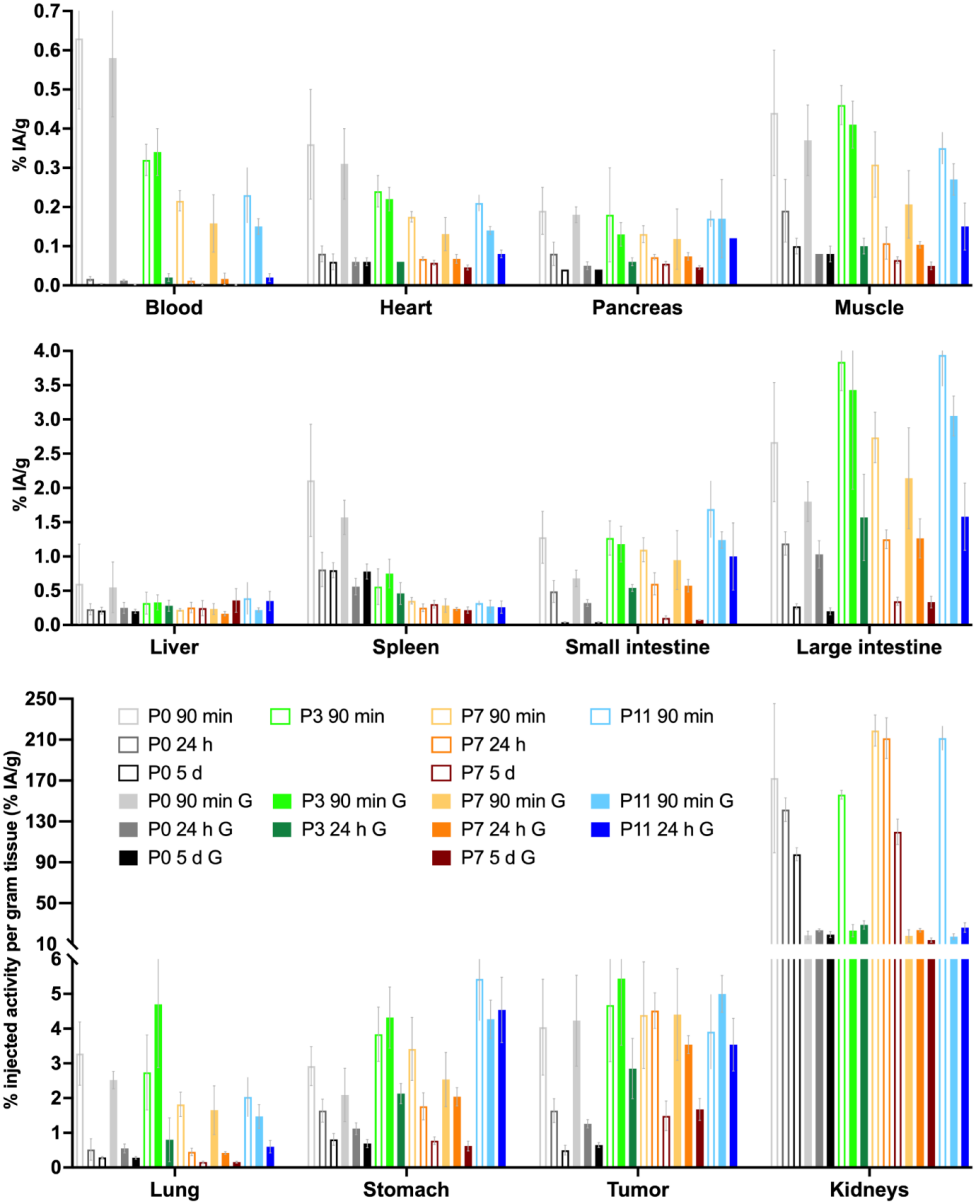
Biodistribution of ^177^Lu-labeled conjugates **P0**, **P3**, **P7**, and **P11** in H2009 xenografted mice; 90 min, 1 d, and 5 d p.i.; with (full bars) and without (empty bars) coinjection of Gelofusine for kidney protection (100 μL, applied as a bolus 2 min before the radiopharmaceuticals). Data for **P0** were reported previously^45^ and are shown for comparison. For data in numerical form, see Supporting Information, Tables S1–S12.

Of specific interest was the observation that the residual blood pool activity after 90 min decreased with increasing PEG linker length, which is indicative of shorter circulation times, while the presence of PEG linkers led to a several-fold increase in tumor uptake after 24 h (Figure 3). However, more than 7 PEG units in the linker appeared to have no further effect on blood clearance or tumor uptake, as no significant difference between **P7** and **P11** was seen in either respect. In our compound series, PEG7 linkers therefore appeared to represent an optimum for in vivo applications, regardless of the superior uptake kinetics in isolated cells observed for **P11** (Figure 1). Based on these observations, **P7** was recognized as the lead candidate among the compounds under consideration and was further characterized in terms of integrin subtype selectivity, αvβ6-integrin-dependent cytotoxicity, and μSPECT imaging.

**Figure 3:**
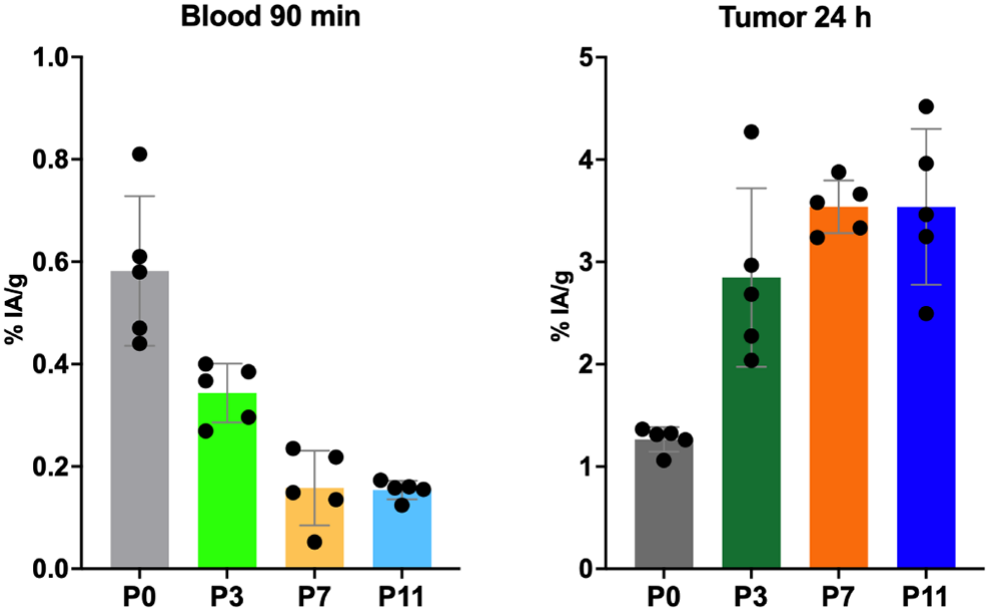
Residual activity in the blood pool (left) and tumor uptake (right) of ^177^Lu-labeled conjugates, 90 min p.i. and 24 h p.i., respectively (*n* = 5).

### Extended Characterization of P7

The affinities of **P7** for the integrins αvβ8, αvβ3, and α5β1 were in the two- to three-digit nanomolar range (Table 2). According to previous experience and literature data,^60^ affinities in this IC_50_ range do not entail a substantial target-specific uptake of integrin-binding radiopharmaceuticals in vivo. αvβ6-integrin selectivity factors ranged from 65 to 671 over the other tested integrins, which suggests that any in vitro or in vivo binding of **P7** is due to specific interaction with αvβ6-integrin, and cross-talk with other integrin subtypes is not a concern.

**Table 2:** Integrin Affinities Determined by ELISA Using the Nonradioactive ^nat^Lu^III^ Equivalent of **P7**, Expressed as 50% Inhibition Constants (IC_50_), and Calculated Integrin Subtype Selectivities for **P7** ^a^

Integrin	IC_50_ (nM)	95% CI (nM)	αvβ6-selectivity
**αvβ6**	0.17	0.09–0.23	–
**αvβ8**	11	4–28	65
**αvβ3**	47	21–117	276
**α5β1**	114	92–143	671

a Selectivities were calculated by dividing the IC_50_ of the respective integrin by the IC_50_ toward αvβ6-integrin. For the respective sigmoidal fit curves, see Supporting Information, Figure S1.

Radiation-induced cytotoxicity was determined using the colorimetric resazurin (alamar blue) assay, which is suitable to quantify cell viability after irradiation.^48^ Radiotoxicity was assessed for different activities per well (range 0.25–1 MBq) for 12 and 24 h incubation times with the radioactivity, both followed by a washing step and a 72 h growth period (Figure 4). A radiation dose-dependent cell-killing effect was observed for αvβ6-integrin-expressing H2009 cells, as well as for αvβ6-integrin-negative PANC-1 cells after incubation with **P7**. However, a stronger reduction of viability was observed for H2009 cells due to αvβ6-integrin-specific binding and internalization of **P7**. In contrast, PANC-1 cells were only irradiated by the evenly distributed radionuclide in the supernatant, resulting in a lower degree of dose-dependent viability reduction. In all experiments, a higher radiation-related cytotoxicity was observed for αvβ6-integrin-positive H2009 cells.

**Figure 4:**
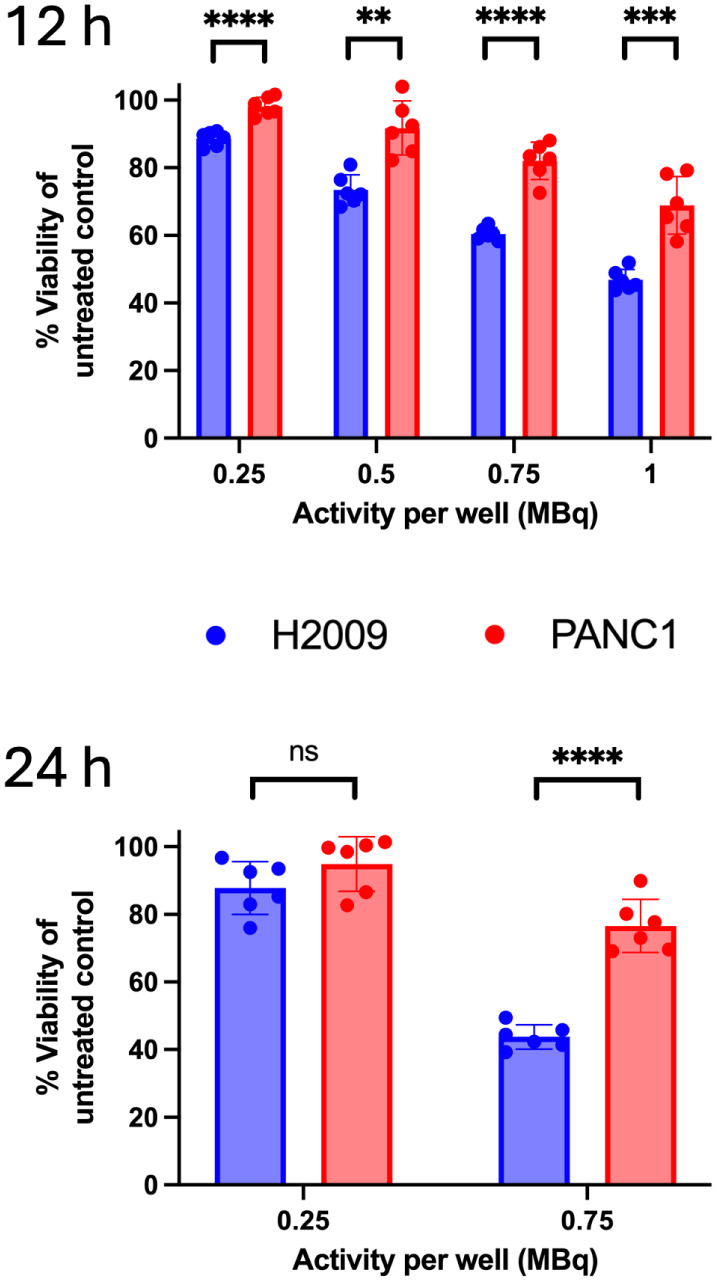
Percent viability of αvβ6-integrin-expressing H2009 cells (blue bars) and αvβ6-integrin-negative PANC-1 cells (red bars), determined using the resazurin (alamar blue) assay^48^ after incubation with different activities of **P7** over 12 h (top) and 24 h (bottom). ** denotes *p* < 0.05; ***, *p* < 0.01; ****, *p* < 0.005; ns, not significant; *n* = 6.

μSPECT images confirmed the overall low background uptake and revealed a tumor retention of **P7** for up to 6 days (Figure 5A). The images also show diffuse abdominal signals originating from the stomach and intestine walls. Furthermore, the comparably high kidney retention causes prominent signals for all time points, which are primarily caused by a long-term retention in the renal cortex (Figure 5B).

**Figure 5:**
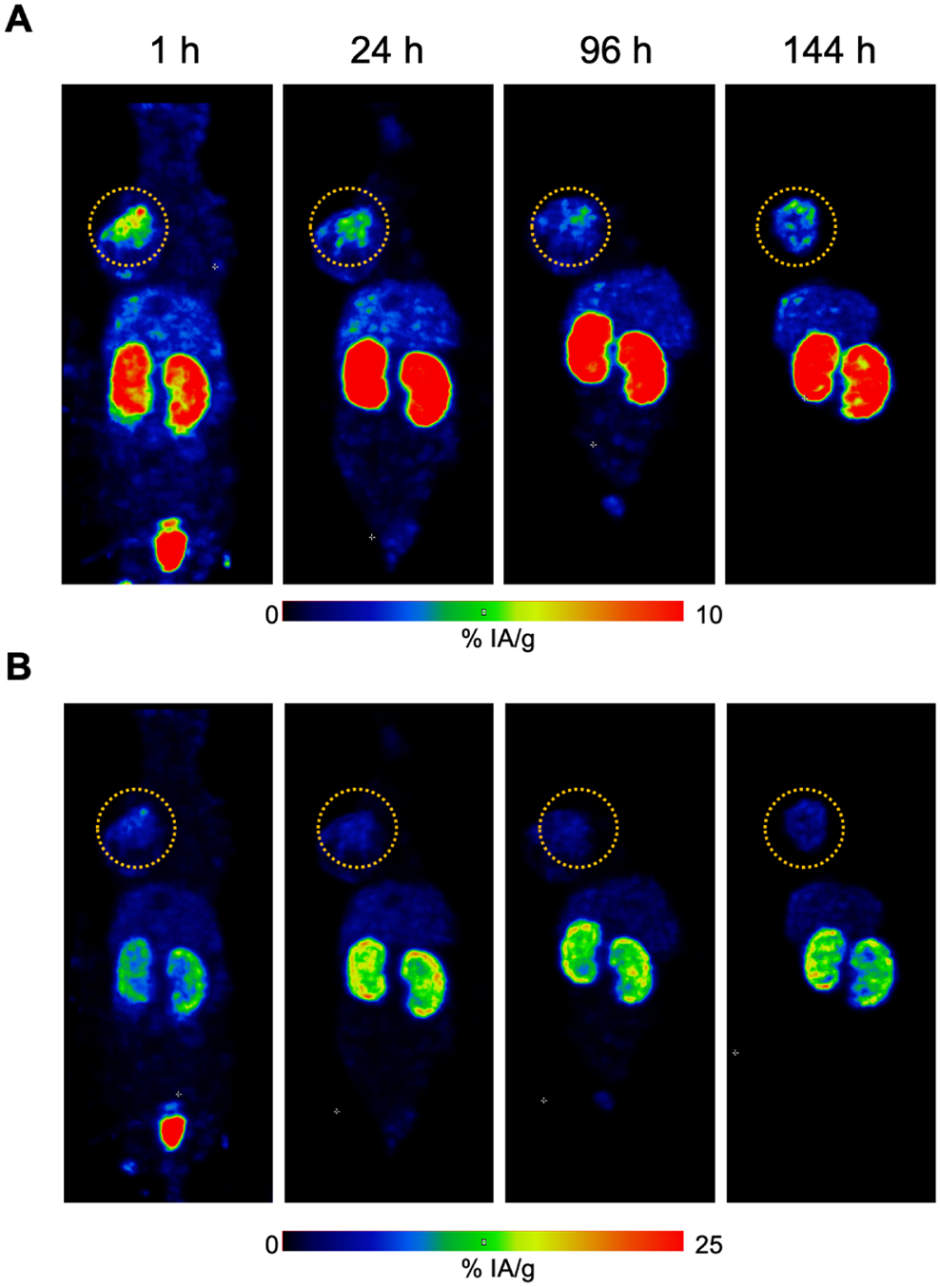
μSPECT images (maximum intensity projections, MIP), recorded 1, 24, 96, and 144 h after administration of 26 MBq of P7 with gelofusine coinjection for kidney protection (100 μL as a bolus 2 min before the activity). (A) Scaled to the tumor. (B) Scaled to the kidneys.

## Discussion

PEGylation is an established method to facilitate clearance from the blood pool via renal excretion and generally to improve the pharmacokinetic properties of drugs.^61^ Attachment of short PEG chains may sharply increase the hydrophilicity of lipophilic compounds, e.g., from log *D* in the range of 1–2 to values near or below zero using PEG4–8 linkers,^62^ but not beyond a certain hydrophilicity threshold. Peptide conjugates with short- or medium-sized PEG linkers typically exhibited a log *D* of −2 to −2.5,^63,64^ which is in line with the values observed for our conjugates. In terms of αvβ6-integrin radiopharmaceuticals, PEG modification has helped to reduce liver accumulation and nonspecific background uptake of αvβ6-targeting cyclopeptides.^59^ For the αvβ6-binding linear peptide A20FMDV, two PEG28 linkers improved the pharmacokinetic profile,^65^ but a shorter PEG5 linker on the *N*-terminus resulted in optimal serum stability and αvβ6-integrin affinity of this inherently metabolically unstable peptide.^66^

For our series of compounds, the presence and length of PEG linkers neither had a strong influence on the hydrophilicity (log *D*) nor on the αvβ6-integrin affinities (IC_50_) determined by competitive ELISA. The log *D* values were found in a narrow range of −1.9 to −2.1, and the IC_50_ ranged from 0.2 to 0.5 nM (rounded), which must be considered similar in view of the limited accuracy of the method. Judging solely on the basis of these parameters, no significantly different behavior of the tested compounds in cell assays or in vivo experiments would have been expected.

Notwithstanding this, we found that PEG linkers of ascending length had a pronounced effect on cellular uptake kinetics. Mechanistically, we assume that linkers with a certain minimal length promote multivalent binding by providing sufficient space between the peptide units, which has been proposed for other RGD-binding integrins before.^67,68^ Multivalent binding interactions to clustered integrins are vastly different from monovalent interactions, strongly affecting signaling and internalization behavior.^69^ Direct evidence of increased internalization^68^ and lysosomal retention^70^ of polyvalent integrin-targeting (radio-)­peptides has been reported but remains scarce. Our cellular uptake data strongly suggest that the PEG linkers in **P3**, and particularly in **P7** and **P11**, promoted multivalent interactions with the αvβ6-integrin, as evidenced by increased intracellular uptake and retention. The optimal distance between peptide units for multivalent binding to αvβ6-integrin has not been well-defined experimentally. In our molecular design, a spacer length of of 7 PEG units was apparently sufficient to enable effective multivalent interactions.

In terms of biodistribution, a slightly lower liver uptake was observed in vivo, although the generally low liver accumulation of Tyr2-based radiopharmaceuticals^28,44,57^ left little room for improvement. Above all, the introduction of PEG linkers showed the desired effect of lower blood pool radioactivity after 90 min, despite an essentially unchanged hydrophilicity according to log *D* measurements. Given the faster blood clearance and shorter circulation time, the approximately three times higher tumor uptake of **P7** and **P11** compared to **P0** was particularly noteworthy because a shorter residence time in the blood pool is usually associated with lower target engagement, i.e., lower tumor uptake.^71,72^ In the present case, accelerated blood clearance was apparently overcompensated for by more efficient target binding and internalization. This assumption is supported by the observation that **P7** showed substantial tumor retention over a period of at least 6 days, which strongly suggests a highly effective trapping in lysosomes. Since the step from 7 to 11 PEG units did not further improve the in vivo performance, **P7** was the preferred lead structure because of its lower molecular weight and better synthetic accessibility of the respective chelator building block, DOTPI-PEG7-triazide.

For all investigated compounds, a strong reduction of the kidney retention (range 156–219%IA/g) could be achieved by gelofusine coinjection, with a maximum of 92% reduction observed for **P7** at 90 min p.i. This observation well aligns with a previous study that highlighted an extraordinarily high efficiency of succinylated gelatin for this compound class as compared to other established kidney protectants, such as arginine/lysine.^27^

In summary, the introduction of PEG3, −7, and −11 linkers into ^177^Lu-labeled trimers of αvβ6-integrin-binding cyclopeptides substantially improved the αvβ6-integrin-mediated internalization into tumor cells, increased tumor uptake in tumor-bearing mice, and accelerated clearance from the blood pool. A linker length of 7 PEG units proved to be optimal, as no further improvements were observed using a longer PEG-11 chain. In conclusion, PEG linkers were successfully employed to optimize the most important pharmacokinetic parameters of ^177^Lu-labeled αvβ6-integrin radiopharmaceuticals for cancer therapy.

## Supplementary Material



## Notes

Synthesis protocols and characterization of DOTPI-PEG3-triazide, DOTPI-PEG7-triazide, DOTPI-PEG11-triazide, ^177^Lu-DOTPI­(PEG3-Tyr2)_3_, ^177^Lu-DOTPI­(PEG7-Tyr2)_3_, and ^177^Lu-DOTPI­(PEG11-Tyr2)_3_; individual curves for uptake kinetics of ^177^Lu-labeled conjugates; sigmoidal fit curves for IC_50_ determination; tables with biodistribution data for ^177^Lu labeled compounds; and a table with experimental details for ELISA (PDF)
