# Supplementary material for: Linker Optimization in Lu-177 Labeled αvβ6-Integrin Binding Peptide Trimers for Targeted Radionuclide Therapy of Cancer
Source: Mol Pharm. 2026 May 27;23(7):3779–88. doi: 10.1021/acs.molpharmaceut.6c00256 (PMC13343512; doi:10.1021/acs.molpharmaceut.6c00256)
Supplement: Supplementary file 1 [file mp6c00256_si_001.pdf]

Supporting Information

Linker optimization in Lu-177 labeled αvβ6-integrin binding peptide trimers for targeted radionuclide therapy of cancer

Nghia Trong Nguyen, Tim Rheinfrank, Stefan Stangl, Falco Reissig,

Susanne Kossatz, Johannes Notni

1 *Department of Nuclear Medicine, TUM University Hospital and Central Institute for Translational Cancer Research, (TranslaTUM), School of Medicine, Technical University Munich, Munich, Germany*

2 *TRIMT GmbH, Carl-Eschebach-Str. 7, 01454 Radeberg, Germany*

3 *Institute of Pathology, School of Medicine, Technical University Munich, Trogerstr. 18, D-81675 München, Germany*

Syntheses

DOTPI-PEG3-triazide

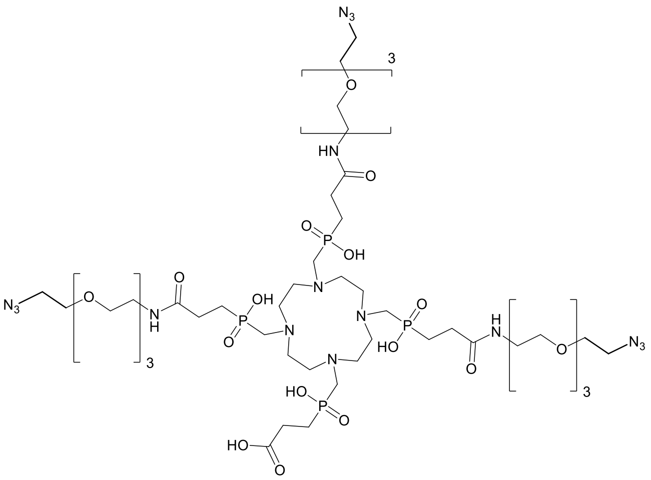

Chemical Formula: $C_{48}H_{96}N_{16}O_{22}P_4$
Molecular Weight: 1373,28

DOTPI • 0.5 H_2O (1.0 eq) and H_2N-PEG3-N_3 (4.0 eq) were dissolved in a dry mixture of DMSO and DIPEA (12.0 eq). HATU (6.0 eq) was added as the last component under stirring. The reaction progress was monitored with HPLC-ESI-MS. After 1 h, the reaction was quenched with water and the volatiles removed under reduced pressure. The crude material was redissolved in H_2O/MeCN and purified via semipreparative RP-HPLC (5–50% H_2O/MeCN + 0.1% TFA, 30 min). Lyophilization yielded the desired product as a colorless solid (yield: 28.0%). Analytical RP-HPLC: (10→90% MeCN + 0.1% TFA, 15 min): t_R= 6.0 min. MS (ESI, positive): m/z = 687.5 [$M+3H^+$], 1373.9 [$M+2H^+$].

DOTPI-PEG7-triazide

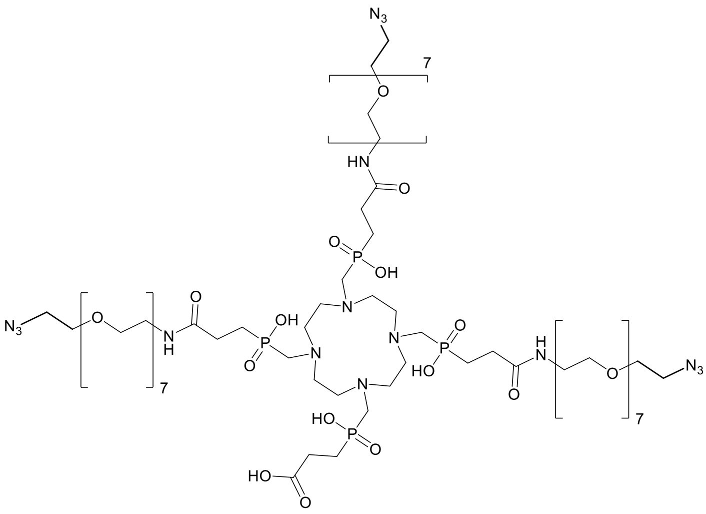

Chemical Formula: $C_{72}H_{144}N_{16}O_{34}P_4$
Molecular Weight: 1901,92

DOTPI • 0.5 H_2O (1.0 eq) and H_2N-PEG7-N_3 (3.5 eq) were dissolved in a dry mixture of DMSO and DIPEA (30.0 eq). PyAOP (10.0 eq) was added as the last component under stirring. The reaction progress was monitored with HPLC-ESI-MS. After 1 h, the reaction was quenched with water and the volatiles removed under reduced pressure. The crude material was redissolved in H_2O/MeCN and purified via semipreparative RP-HPLC (20–45% H_2O/MeCN + 0.1% TFA, 30 min). Lyophilization yielded the desired product as a yellow oil (yield: 18.9%). Analytical RP-HPLC: (10→90% MeCN + 0.1% TFA, 15 min): t_R= 6.2 min. MS (ESI, positive): m/z = 951.6 [M+2H$^+$].

DOTPI-PEG11-triazide

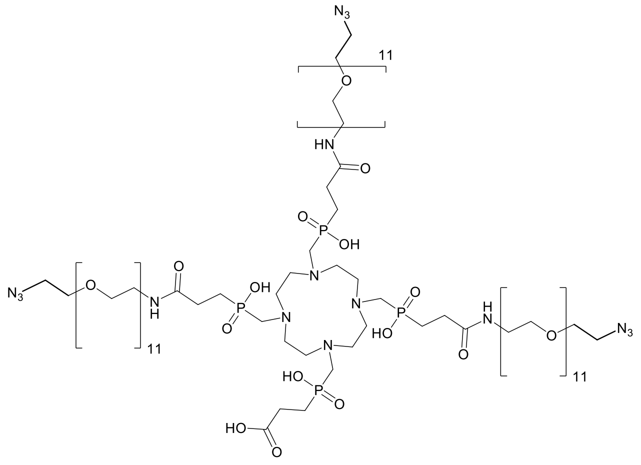

Chemical Formula: $C_{96}H_{192}N_{16}O_{46}P_4$
Molecular Weight: 2430,55

DOTPI • 0.5 H_2O (1.0 eq) and H_2N-PEG11-N_3 (3.7 eq) were dissolved in a dry mixture of DMSO and DIPEA (30.0 eq). PyAOP (10.0 eq) was added as the last component under stirring. The reaction progress was monitored with HPLC-ESI-MS. After 1 h the reaction was quenched with water and the volatiles removed under reduced pressure. The crude material was redissolved in H_2O/MeCN and purified via semipreparative RP-HPLC (35–44% H_2O/MeCN + 0.1% TFA, 30 min). Lyophilization yielded the desired product as a yellow oil (yield: 11.1%). Analytical RP-HPLC: (35→55% MeCN + 0.1% TFA, 15 min): t_R= 8.3 min. MS (ESI, positive): m/z = 816.9 [M+Na$^+$+2H$^+$], 1215.9 [M+2H$^+$].

DOTPI(PEG3-Tyr2)$_3$

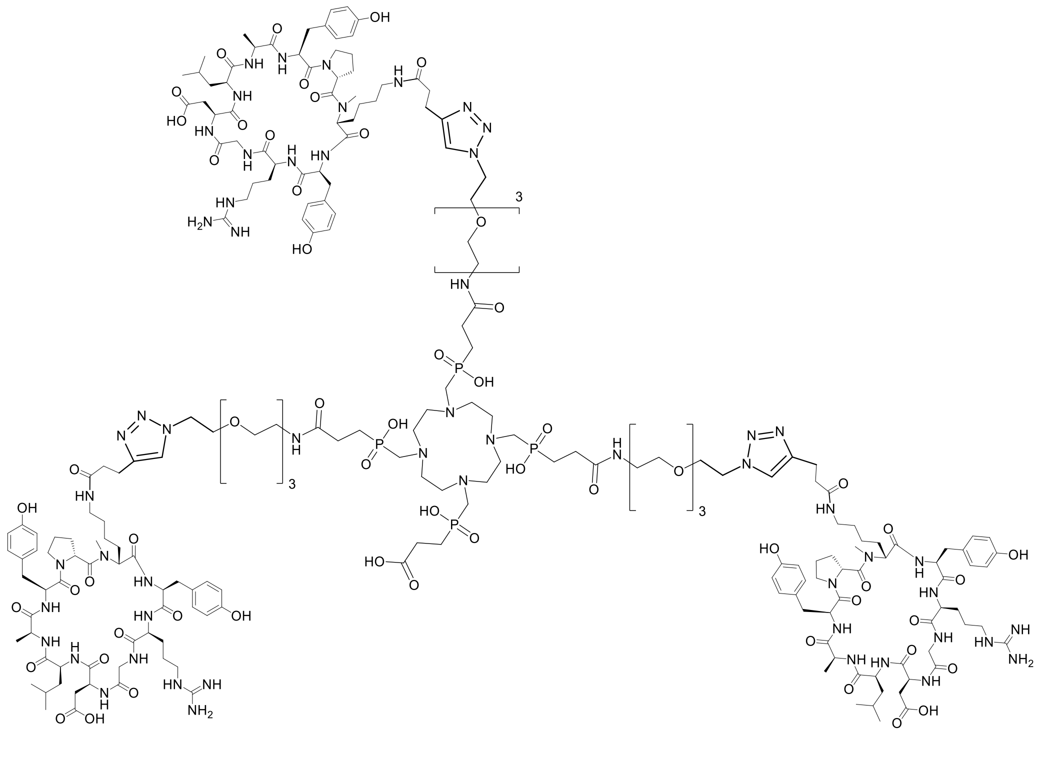

Chemical Formula: $C_{216}H_{333}N_{55}O_{64}P_4$
Molecular Weight: 4848,26

DOTPI-PEG3-triazide (1.0 eq), Tyr2-alkyne (3.7 eq) and sodium ascorbate (50.0 eq) were dissolved in H_2O/*t*BuOH (1:1). The reaction was started by adding an aqueous solution of $Cu(OAc)_2$ monohydrate (2.0 eq) while stirring, which resulted in a brown precipitate that dissolved after stirring for several seconds. The reaction progress and copper transchelation was monitored with HPLC-ESI-MS. After 30 min, the reaction was quenched by the addition of an aqueous solution of NOTA (35.0 eq). The mixture was stirred at room temperature overnight at pH 2.2. Then, the solvents were removed under reduced pressure, the crude product was redissolved in H_2O/MeCN, and purified via semipreparative RP-HPLC (5–50% H_2O/MeCN + 0.1% TFA, 30 min). Lyophilization yielded the desired product as a colorless solid (yield: 6.0%). Analytical RP-HPLC: (10→90% MeCN + 0.1% TFA, 15 min): t_R= 9.0 min. MS (ESI, positive): m/z = 970.6 $[M+5H^+]$, 1231.4 $[M+4H^+]$, 1616.9 $[M+3H^+]$.

DOTPI(PEG7-Tyr2)$_3$

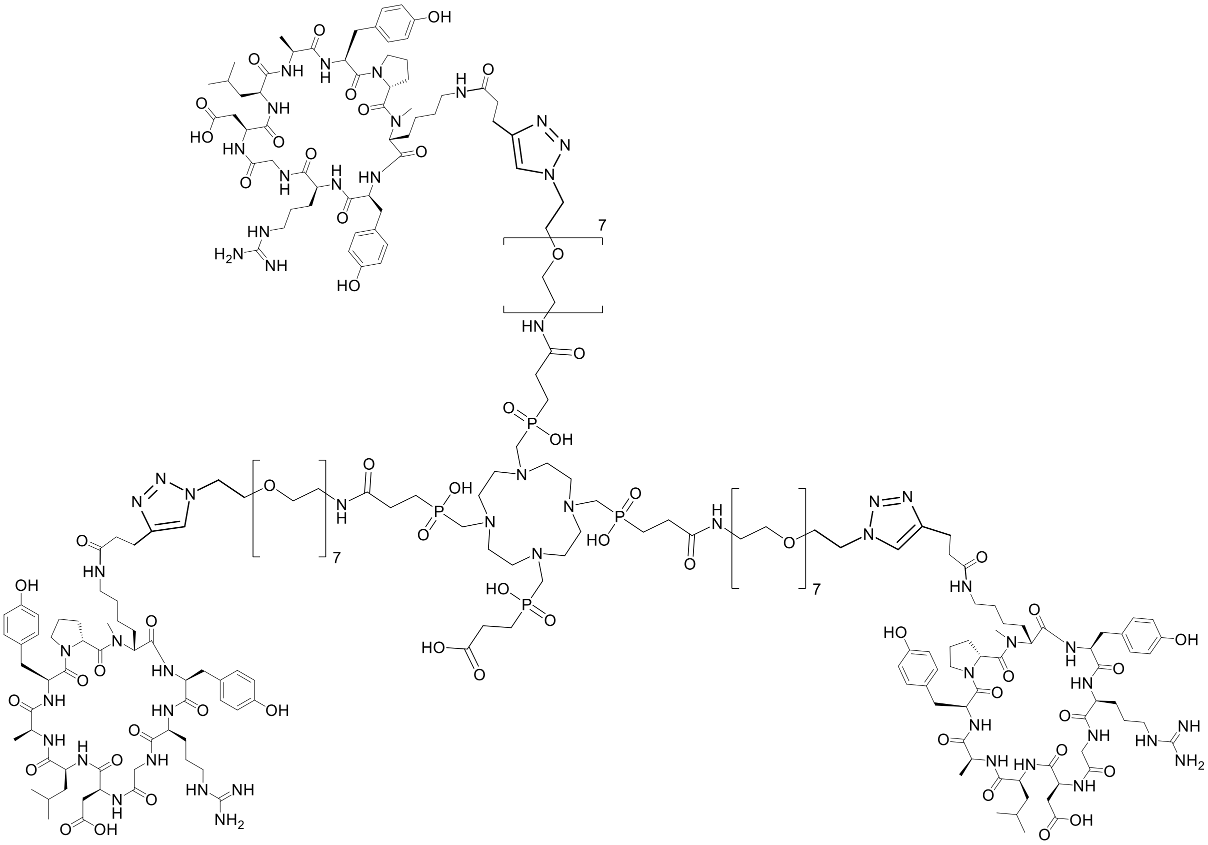

Chemical Formula: $C_{240}H_{381}N_{55}O_{76}P_4$
Molecular Weight: 5376,89

DOTPI-PEG7-triazide (1.0 eq), Tyr2-alkyne (3.3 eq) and sodium ascorbate (50.0 eq) were dissolved in H_2O/*t*BuOH (1:1). The reaction was started by adding an aqueous solution of $Cu(OAc)_2$ monohydrate (2.0 eq) while stirring, which resulted in a brown precipitate that dissolved after stirring for several seconds. The reaction progress and copper transchelation was monitored with HPLC-ESI-MS. After 30 min, the reaction was quenched by the addition of an aqueous solution of NOTA (35.0 eq). The mixture was stirred at room temperature overnight at pH 2.2. Then, the solvents were removed under reduced pressure, the crude product was redissolved in H_2O/MeCN, and purified via semipreparative RP-HPLC (25–38% H_2O/MeCN + 0.1% TFA, 30 min). Lyophilization yielded the desired product as a light blue solid (yield: 12.3%). Analytical RP-HPLC: (25→40% MeCN + 0.1% TFA, 15 min): t_R= 11.0 min. MS (ESI, positive): m/z = 897.3 [$M+6H^+$], 1076.5 [$M+5H^+$], 1345.3 [$M+4H^+$].

DOTPI(PEG11-Tyr2)$_3$

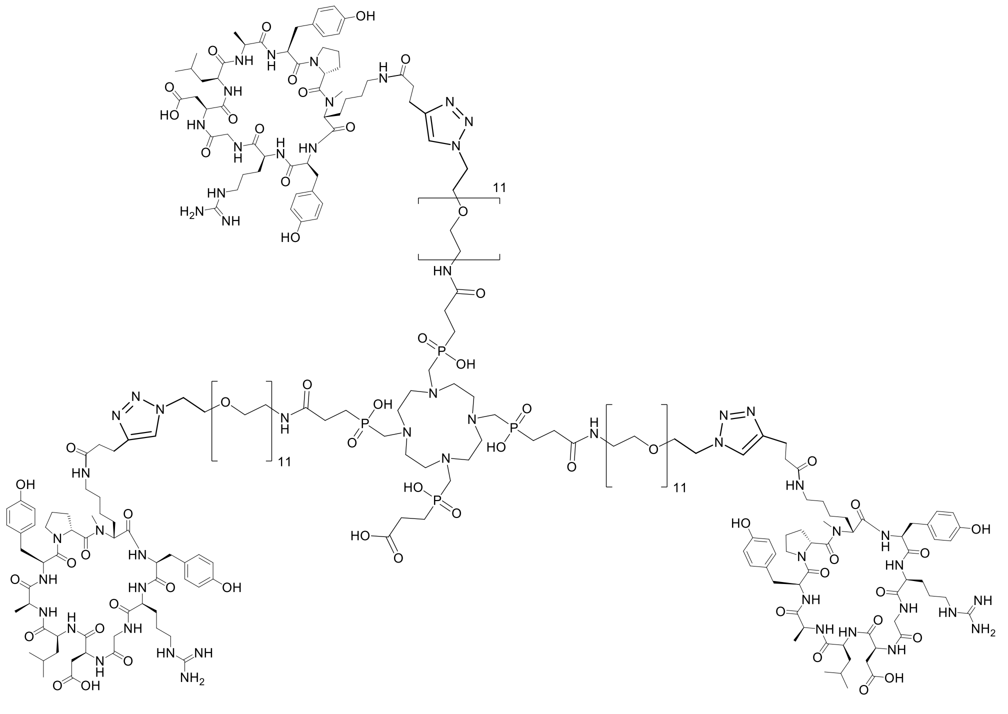

Chemical Formula: $C_{264}H_{429}N_{55}O_{88}P_4$
Molecular Weight: 5905,53

DOTPI-PEG11-triazide (1.0 eq), Tyr2-alkyne (3.3 eq) and sodium ascorbate (50.0 eq) were dissolved in H_2O/*t*BuOH (1:1). The reaction was started by adding an aqueous solution of $Cu(OAc)_2$ monohydrate (2.0 eq) while stirring, which resulted in a brown precipitate that dissolved after stirring for several seconds. The reaction progress and copper transchelation was monitored with HPLC-ESI-MS. After 30 min, the reaction was quenched by the addition of an aqueous solution of NOTA (35.0 eq). The mixture was stirred at room temperature overnight at pH 2.2. Then, the solvents were removed under reduced pressure, the crude product was redissolved in H_2O/MeCN, and purified via semipreparative RP-HPLC (25–40% H_2O/MeCN + 0.1% TFA, 30 min). Lyophilization yielded the desired product as a light blue solid (yield: 28.2%). Analytical RP-HPLC: (10→90% MeCN + 0.1% TFA, 15 min): t_R= 7.3 min. MS (ESI, positive): m/z = 844.8 [M+7H$^+$], 1182.0 [M+5H$^+$], 1477.3 [M+4H$^+$].

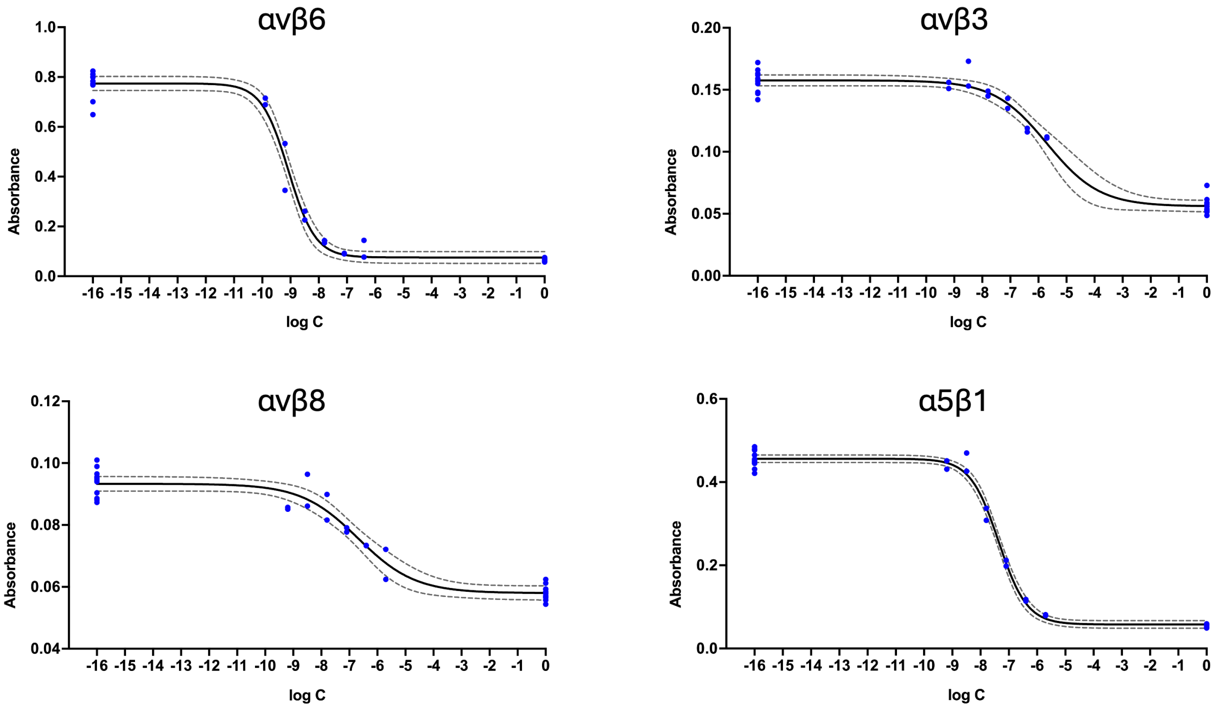

Figure S1: Sigmoidal fit curves for calculation of integrin binding affinities (IC_{50} values) for **P7** from ELISA data.

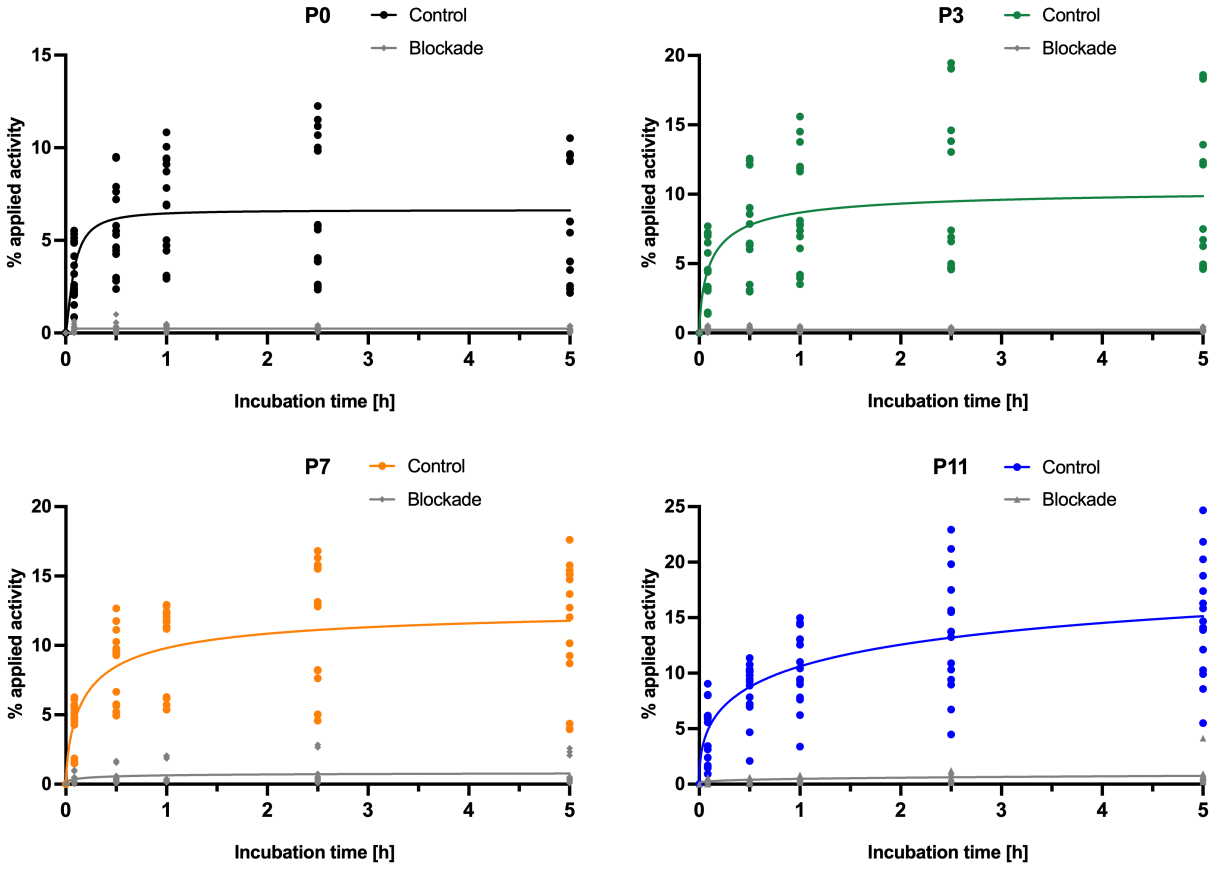

Figure S2: Uptake kinetics of ^{177}Lu-labeled conjugates (0.3 nM, 50 MBq/nmol) in H2009 cells, blockade with 250 nM ^{nat}Ga-Trivehexin; 4-parameter variable slope fit curves to data acquired for incubation times of 5 min, 30 min, 1 h, 2.5 h, and 5 h, at 37 °C; n=15 per time point (5 biological repeats à n=3).

Biodistribution data

Table S1: Biodistribution data for **P3** (*n* = 4; 66.4 ± 14.8 pmol), in H2009 bearing SCID mice, 90 min p.i.. Data are given as averages ± standard deviation. %IA/g = percent injected activity per gram tissue.

**P3**	**90 min p.i.**					
Organ/Tissue	%IA/g			tumor/organ ratio		
Blood	0.32	±	0.04	14.4	±	4.5
Heart (myocard)	0.24	±	0.04	20.2	±	9.5
Lung	2.74	±	1.08	1.8	±	0.6
Liver	0.32	±	0.16	15.7	±	4.4
Spleen	0.56	±	0.26	9.6	±	5.6
Pancreas	0.18	±	0.12	38	±	28
Stomach (empty)	3.8	±	0.8	1.2	±	0.4
Small intestine (empty)	1.27	±	0.25	3.7	±	1.3
Large intestine (empty)	3.8	±	0.4	1.2	±	0.5
Muscle	0.46	±	0.05	10.6	±	4.3
Tumor H2009	4.68	±	1.63		–	
Kidneys	156.1	±	4.3	0.030	±	0.010

Table S2: Biodistribution data for **P3** (*n* = 5; 39.7 ± 18.9 pmol), in H2009 bearing SCID mice, 90 min p.i., with co-infusion of Gelofusine (100 µL, 2 min before the activity). Data are given as averages ± standard deviation. %IA/g = percent injected activity per gram tissue.

**P3**	**90 min p.i. + Gelofusine**					
Organ/Tissue	%IA/g			tumor/organ ratio		
Blood	0.34	±	0.06	16.2	±	5.8
Heart (myocard)	0.22	±	0.03	24.1	±	7.1
Lung	4.7	±	1.82	1.2	±	0.5
Liver	0.33	±	0.11	18.2	±	8.4
Spleen	0.75	±	0.21	7.4	±	2.7
Pancreas	0.13	±	0.03	41	±	10
Stomach (empty)	4.3	±	0.9	1.3	±	0.6
Small intestine (empty)	1.18	±	0.26	4.9	±	1.9
Large intestine (empty)	3.4	±	1.5	1.8	±	0.5
Muscle	0.41	±	0.06	14.0	±	6.1
Tumor H2009	5.4	±	1.9		–	
Kidneys	23.2	±	6.1	0.244	±	0.100

Table S3: Biodistribution data for **P3** (*n* = 5; 43.3 ± 8.4 pmol), in H2009 bearing SCID mice, 24 h p.i., with co-infusion of Gelofusine (100 µL, 2 min before the activity). Data are given as averages ± standard deviation. %IA/g = percent injected activity per gram tissue.

**P3**	**24 h p.i. + Gelofusine**	
Organ/Tissue	%IA/g	tumor/organ ratio
Blood	0.02 ± 0.01	189 ± 49
Heart (myocard)	0.062 ± 0.004	46 ± 14
Lung	0.8 ± 0.63	4.7 ± 2.2
Liver	0.28 ± 0.08	10.3 ± 2.4
Spleen	0.46 ± 0.16	7.0 ± 4.2
Pancreas	0.06 ± 0.01	49 ± 19
Stomach (empty)	2.13 ± 0.29	1.4 ± 0.4
Small intestine (empty)	0.54 ± 0.05	5.3 ± 1.3
Large intestine (empty)	1.57 ± 0.63	2.0 ± 0.7
Muscle	0.1 ± 0.02	29 ± 12
Tumor H2009	2.85 ± 0.87	–
Kidneys	28.8 ± 4.1	0.100 ± 0.031

Table S4: Biodistribution data for **P7** (*n* = 4; 44.1 ± 5.5 pmol), in H2009 bearing SCID mice, 90 min p.i.. Data are given as averages ± standard deviation. %IA/g = percent injected activity per gram tissue.

**P7**	**90 min p.i.**	
Organ/Tissue	%IA/g	tumor/organ ratio
Blood	0.22 ± 0.03	21.0 ± 9.0
Heart (myocard)	0.18 ± 0.01	25.2 ± 8.7
Lung	1.82 ± 0.35	2.4 ± 0.6
Liver	0.22 ± 0.02	20.2 ± 8.0
Spleen	0.35 ± 0.05	12.8 ± 5.4
Pancreas	0.13 ± 0.02	35 ± 14
Stomach (empty)	3.4 ± 0.9	1.4 ± 0.5
Small intestine (empty)	1.09 ± 0.17	4.1 ± 1.5
Large intestine (empty)	2.7 ± 0.4	1.6 ± 0.5
Muscle	0.31 ± 0.08	14.9 ± 5.8
Tumor H2009	4.4 ± 1.5	–
Kidneys	219 ± 15	0.020 ± 0.006

Table S5: Biodistribution data for **P7** (*n* = 4; 74.7 ± 14.2 pmol), in H2009 bearing SCID mice, 24 h p.i.. Data are given as averages ± standard deviation. %IA/g = percent injected activity per gram tissue.

**P7**	**24 h p.i.**					
Organ/Tissue	%IA/g			tumor/organ ratio		
Blood	0.012	±	0.007	460	±	177
Heart (myocard)	0.068	±	0.005	67	±	12
Lung	0.45	±	0.11	10.5	±	2.9
Liver	0.26	±	0.08	18.6	±	4.2
Spleen	0.25	±	0.06	18.3	±	2.4
Pancreas	0.072	±	0.007	63	±	6
Stomach (empty)	1.76	±	0.39	2.6	±	0.6
Small intestine (empty)	0.60	±	0.16	7.8	±	1.7
Large intestine (empty)	1.25	±	0.14	3.7	±	0.7
Muscle	0.11	±	0.04	45	±	12
Tumor H2009	4.5	±	0.51		–	
Kidneys	211	±	20	0.021	±	0.002

Table S6: Biodistribution data for **P7** (*n* =4 ; 85.5 ± 4.8 pmol), in H2009 bearing SCID mice, 5 d p.i.. Data are given as averages ± standard deviation. %IA/g = percent injected activity per gram tissue.

**P7**	**5 d p.i.**					
Organ/Tissue	%IA/g			tumor/organ ratio		
Blood	0.0029	±	0.003	746	±	393
Heart (myocard)	0.058	±	0.006	27	±	7.5
Lung	0.157	±	0.03	9.5	±	2.3
Liver	0.246	±	0.111	6.9	±	3.7
Spleen	0.30	±	0.06	4.9	±	1.0
Pancreas	0.056	±	0.006	27	±	6
Stomach (empty)	0.77	±	0.11	1.9	±	0.5
Small intestine (empty)	0.103	±	0.029	15.0	±	5.4
Large intestine (empty)	0.35	±	0.06	4.4	±	1.5
Muscle	0.065	±	0.009	22.8	±	4.4
Tumor H2009	1.49	±	0.43		–	
Kidneys	120	±	12	0.012	±	0.003

Table S7: Biodistribution data for **P7** (*n* = 5; 53.4 ± 29.0 pmol), in H2009 bearing SCID mice, 90 min p.i., with co-infusion of Gelofusine (100 µL, 2 min before the activity). Data are given as averages ± standard deviation. %IA/g = percent injected activity per gram tissue.

**P7**	**90 min p.i. + Gelofusine**	
Organ/Tissue	%IA/g	tumor/organ ratio
Blood	0.16 ± 0.07	29 ± 12
Heart (myocard)	0.13 ± 0.04	32 ± 7
Lung	1.65 ± 0.70	2.7 ± 0.9
Liver	0.23 ± 0.08	18.1 ± 4.1
Spleen	0.28 ± 0.10	14.8 ± 3.0
Pancreas	0.12 ± 0.08	43 ± 17
Stomach (empty)	2.54 ± 0.78	1.6 ± 0.3
Small intestine (empty)	0.95 ± 0.43	4.8 ± 1.6
Large intestine (empty)	2.14 ± 0.74	2.0 ± 0.5
Muscle	0.21 ± 0.09	21.5 ± 6.8
Tumor H2009	4.4 ± 1.3	–
Kidneys	17.9 ± 6.1	0.236 ± 0.056

Table S8: Biodistribution data for **P7** (*n* = 5; 53.0 ± 3.6 pmol), in H2009 bearing SCID mice, 24 h p.i., with co-infusion of Gelofusine (100 µL, 2 min before the activity). Data are given as averages ± standard deviation. %IA/g = percent injected activity per gram tissue.

**P7**	**24 h p.i. + Gelofusine**	
Organ/Tissue	%IA/g	tumor/organ ratio
Blood	0.017 ± 0.015	440 ± 321
Heart (myocard)	0.068 ± 0.011	53 ± 6
Lung	0.42 ± 0.038	8.6 ± 1.3
Liver	0.16 ± 0.032	22.1 ± 3.6
Spleen	0.23 ± 0.023	15.4 ± 1.7
Pancreas	0.074 ± 0.001	48 ± 4
Stomach (empty)	2.04 ± 0.26	1.8 ± 0.1
Small intestine (empty)	0.57 ± 0.09	6.3 ± 0.7
Large intestine (empty)	1.26 ± 0.29	2.9 ± 0.7
Muscle	0.104 ± 0.008	34 ± 2
Tumor H2009	3.54 ± 0.26	–
Kidneys	23.6 ± 1.8	0.151 ± 0.011

Table S9: Biodistribution data for **P7** (*n* = 5; 96.0 ± 13.2 pmol), in H2009 bearing SCID mice, 5 d p.i., with co-infusion of Gelofusine (100 µL, 2 min before the activity). Data are given as averages ± standard deviation. %IA/g = percent injected activity per gram tissue.

**P7**	**5 d p.i. + Gelofusine**					
Organ/Tissue	%IA/g			tumor/organ ratio		
Blood	0.0014	±	0.0011	2515	±	2187
Heart (myocard)	0.046	±	0.006	40	±	7
Lung	0.156	±	0.024	11.5	±	1.1
Liver	0.36	±	0.173	5.7	±	2.1
Spleen	0.22	±	0.045	8.7	±	3.5
Pancreas	0.044	±	0.004	39	±	6
Stomach (empty)	0.62	±	0.14	2.9	±	0.4
Small intestine (empty)	0.073	±	0.006	25	±	4
Large intestine (empty)	0.33	±	0.08	5.5	±	0.9
Muscle	0.05	±	0.01	36	±	3
Tumor H2009	1.67	±	0.32		–	
Kidneys	13.9	±	2.1	0.130	±	0.020

Table S10: Biodistribution data for **P11** (*n* = 4; 53.5 ± 4.2 pmol), in H2009 bearing SCID mice, 90 min p.i.. Data are given as averages ± standard deviation. %IA/g = percent injected activity per gram tissue.

**P11**	**90 min p.i.**					
Organ/Tissue	%IA/g			tumor/organ ratio		
Blood	0.23	±	0.07	18.8	±	10.4
Heart (myocard)	0.21	±	0.02	19.1	±	6.6
Lung	2.03	±	0.56	1.9	±	0.4
Liver	0.39	±	0.23	11.3	±	4.1
Spleen	0.32	±	0.03	12.2	±	2.5
Pancreas	0.17	±	0.02	24	±	8
Stomach (empty)	5.4	±	1.2	0.73	±	0.24
Small intestine (empty)	1.69	±	0.41	2.5	±	0.9
Large intestine (empty)	3.9	±	0.5	0.99	±	0.24
Muscle	0.35	±	0.04	11.5	±	4.0
Tumor H2009	3.9	±	1.1		–	
Kidneys	212	±	12	0.019	±	0.006

Table S11: Biodistribution data for **P11** (*n* = 5; 56.8 ± 7.9 pmol), in H2009 bearing SCID mice, 90 min p.i., with co-infusion of Gelofusine (100 µL, 2 min before the activity). Data are given as averages ± standard deviation. %IA/g = percent injected activity per gram tissue.

**P11**	**90 min p.i. + Gelofusine**	
Organ/Tissue	%IA/g	tumor/organ ratio
Blood	0.15 ± 0.02	33 ± 2
Heart (myocard)	0.14 ± 0.01	36 ± 4
Lung	1.47 ± 0.34	3.5 ± 0.7
Liver	0.22 ± 0.03	23.2 ± 2.3
Spleen	0.27 ± 0.09	20.6 ± 8.0
Pancreas	0.17 ± 0.1	34 ± 12
Stomach (empty)	4.3 ± 0.6	1.19 ± 0.23
Small intestine (empty)	1.24 ± 0.12	4.1 ± 0.5
Large intestine (empty)	3.1 ± 0.3	1.65 ± 0.17
Muscle	0.27 ± 0.04	18.3 ± 0.9
Tumor H2009	5.0 ± 0.5	–
Kidneys	17.3 ± 2.8	0.296 ± 0.066

Table S12: Biodistribution data for **P11** (*n* = 5; 48.1 ± 5.5 pmol), in H2009 bearing SCID mice, 24 h p.i., with co-infusion of Gelofusine (100 µL, 2 min before the activity). Data are given as averages ± standard deviation. %IA/g = percent injected activity per gram tissue.

**P11**	**24 h p.i. + Gelofusine**	
Organ/Tissue	%IA/g	tumor/organ ratio
Blood	0.017 ± 0.006	245 ± 140
Heart (myocard)	0.077 ± 0.014	46 ± 10
Lung	0.60 ± 0.18	6.1 ± 1.4
Liver	0.35 ± 0.14	11.6 ± 4.8
Spleen	0.26 ± 0.09	14.9 ± 5.5
Pancreas	0.119 ± 0.004	30 ± 6
Stomach (empty)	4.5 ± 0.9	0.78 ± 0.05
Small intestine (empty)	1.00 ± 0.49	5.1 ± 4.3
Large intestine (empty)	1.58 ± 0.49	2.37 ± 0.65
Muscle	0.153 ± 0.06	26 ± 10
Tumor H2009	3.5 ± 0.8	–
Kidneys	26.2 ± 4.7	0.136 ± 0.023

Experimental details for ELISA

Table S13: Experimental Details of the ELISA protocol for integrin affinity determination.

Assay	αvβ6	αvβ8	αvβ3	α5β1
**ECM protein**	LAP (TGFβ) (biotechne: 246-LP-025) 0.4 µg/mL	LAP (TGFβ) (biotechne: 246-LP-025) 0.4 µg/mL	human vitronectin (Sigma-Aldrich: F0895) 1.0 µg/mL	human fibronectin (Merck: CC080) 0.5 µg/mL
**Integrin**	human αvβ6-integrin (biotechne: 3817-AV), 0.5 µg/mL	human αvβ8-integrin (biotechne: 4135-AV), , 0.5 µg/mL	human αvβ3-integrin (biotechne: 3050-AV), , 2.0 µg/mL	human α5β1-integrin (biotechne: 3230-AV), , 2.0 µg/mL
**Primary AB**	anti-αv mouse anti-human (Merck: MAB1978), 1:500 dilution	anti-αv mouse anti-human (Merck: MAB1978), 1:500 dilution	mouse anti-human CD51/61 (BD biosciences: 555504), 2.0 µg/mL	mouse anti-human CD49e (BD biosciences: 555651), 1.0 µg/mL
**Secondary AB**	anti-mouse IgG-POD (Sigma-Aldrich: A5906), 2.0 µg/mL	anti-mouse IgG-POD (Sigma-Aldrich: A5906), 2.0 µg/mL	anti-mouse IgG-POD (Sigma-Aldrich: A5906), 1.0 µg/mL	anti-mouse IgG-POD (Sigma-Aldrich: A5906), 2.0 µg/mL
**Standard**	^{nat}Ga-Trivehexin	RTDLDSLRT	Cilengitide	Cilengitide
**Ref. IC_{50}**	0.047 nM	100 nM	0.54 nM	15.4 nM

^{nat}Lu-P0

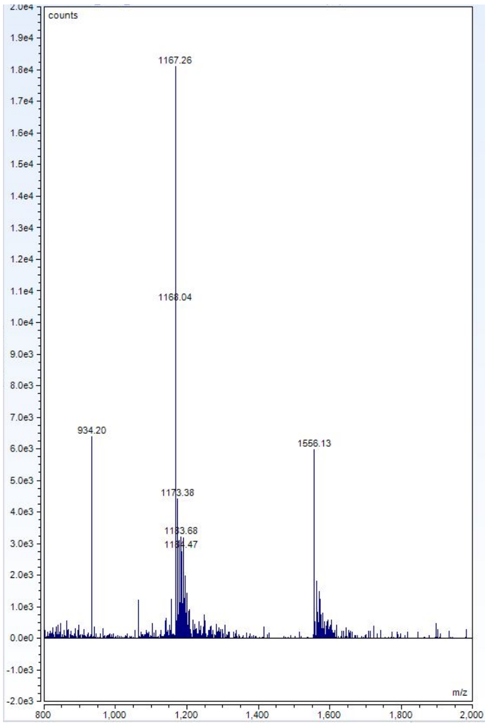

^{nat}Lu-P3

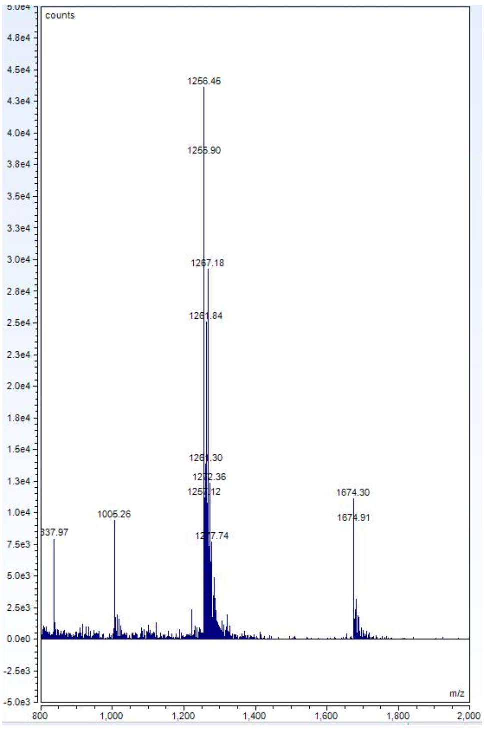

^{nat}Lu-P7

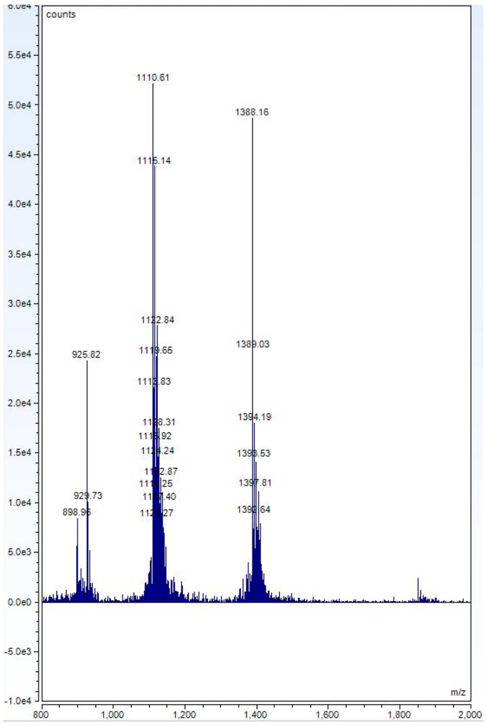

^{nat}Lu-P11

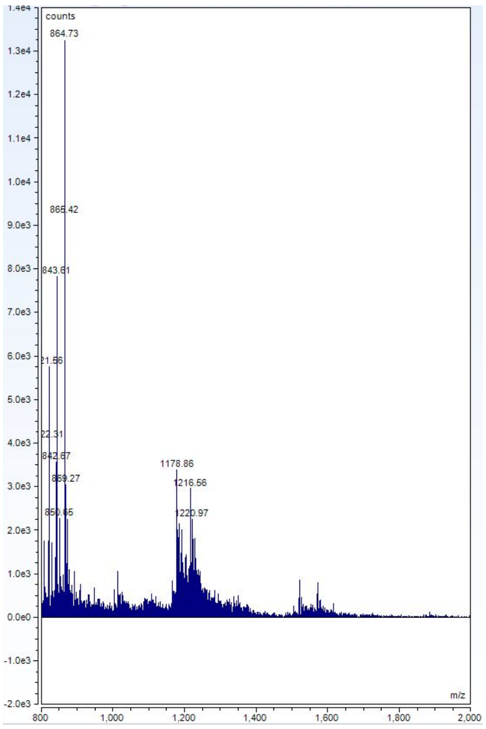

Figure S3: ESI-MS spectra for ^{nat}Lu labeled compounds **P0**, **P3**, **P7**, and **P11**

P0

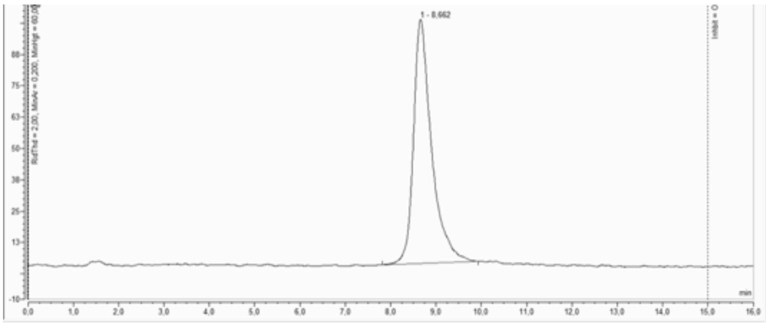

P3

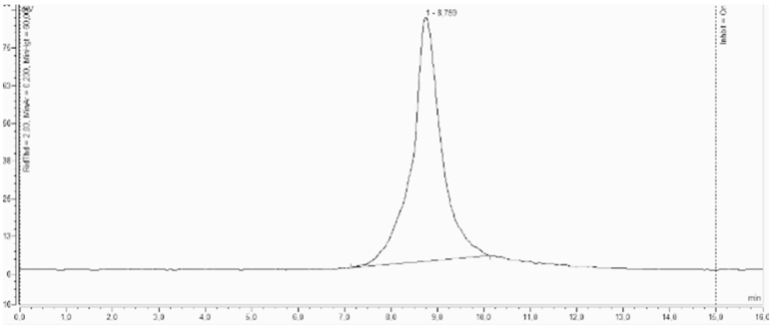

P7

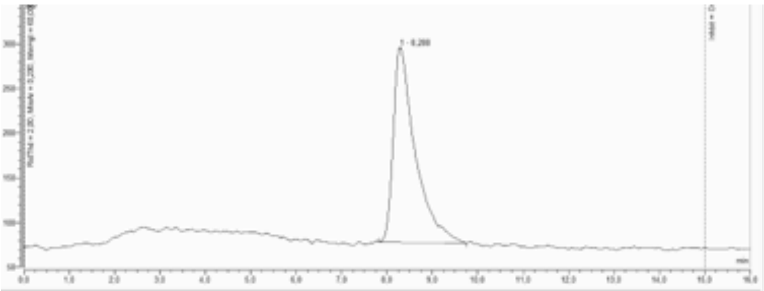

P11

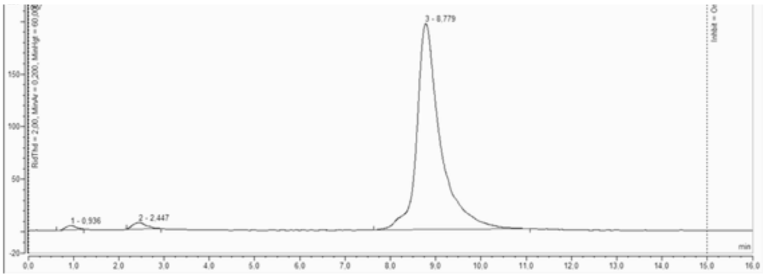

Figure S4: Radio-HPLC chromatograms for compounds **P0**, **P3**, **P7**, and **P11**
